# *Burkholderia pseudomallei* Antibodies in Children, Cambodia

**DOI:** 10.3201/eid1402.070811

**Published:** 2008-02

**Authors:** Vanaporn Wuthiekanun, Ngoun Pheaktra, Hor Putchhat, Lina Sin, Bun Sen, Varun Kumar, Sayan Langla, Sharon J. Peacock, Nicholas P. Day

**Affiliations:** * Mahidol University, Bangkok, Thailand; † Angkor Hospital for Children, Siem Reap, Cambodia; ‡ University of Oxford, Oxford, United Kingdom

**Keywords:** Burkholderia pseudomallei, Cambodia, environmental sampling, melioidosis, seropositivity, dispatch

## Abstract

Antibodies to *Burkholderia pseudomallei* were detected in 16% of children in Siem Reap, Cambodia. This organism was isolated from 30% of rice paddies in the surrounding vicinity. Despite the lack of reported indigenous cases, melioidosis is likely to occur in Cambodia.

*Burkholderia pseudomallei* is a soil saprophyte and the cause of melioidosis (*1*). This bacterium can be isolated from soil and water in melioidosis-endemic regions of the tropics, where infection is acquired after bacterial inoculation, inhalation, or ingestion (*1*). Most reported cases occur in Thailand and northern Australia, but this statistic likely represents a fraction of the true extent of disease because microbial culture, the mainstay of diagnostic confirmation, is not available across much of rural Asia. The largest concentration of confirmed melioidosis cases worldwide occurs in northeast Thailand, where the disease accounts for 20% of all community-acquired septicemias. The death rate for affected adults in this setting is ≈50% (*2*). Northeast Thailand is bordered by Lao People’s Democratic Republic (PDR) to the east and southeast and Cambodia to the south. Melioidosis has recently been recognized in Lao PDR after a diagnostic microbiology laboratory was instituted at Mahosot Hospital, Vientiene (*3*), and *B. pseudomallei* has been isolated from the surrounding environment (*4*). By contrast, there are no reports in the literature of indigenous melioidosis or environmental isolation of *B. pseudomallei* in adjacent Cambodia. Two cases of melioidosis have been reported in Cambodian residents in Canada and the United States, respectively; both persons had spent several years in refugee camps in Thailand (*5*,*6*). We propose that melioidosis occurs in Cambodia but is unrecognized because of the lack of diagnostic microbiology facilities. The aims of this study were to conduct a seroprevalence study of children living in Siem Reap, Cambodia, to detect the presence of antibodies resulting from exposure to *B. pseudomallei*, and to determine whether this organism could be isolated from their environment.

## The Study

A prospective, cross-sectional study was conducted at Angkor Hospital for Children, Siem Reap, from December 2005 through April 2006. Unselected consecutive serum samples were collected from children between birth and 16 years of age from the biochemistry and hematology laboratory of Angkor Hospital for Children, Siem Reap. Blood samples were collected from outpatients and inpatients. These blood tests were ordered by the primary physician for other reasons, and the sample used represented surplus material. Samples were centrifuged at 3,000 rpm for 10 min and the serum stored at –30°C. Target sample numbers were 40–60 per year group. An anonymous database was created to record sex, age, and indirect hemagglutination assay (IHA) titer. The presence and titer of antibodies to *B. pseudomallei* were determined by using the IHA, as previously described (*7*), with the exception that the pooled antigens used were from 2 *B. pseudomallei* isolates (strains 001a and 002a) isolated 10 years ago in Phnom Penh, Cambodia. These patients’ isolates were sent to us for identification in 1996, although the details of their clinical provenance are unknown. Although data on these isolates are unpublished, their existence suggests the presence of melioidosis in Cambodia. Serum samples were tested in random order. Any detectable IHA titer was interpreted as evidence of exposure to *B. pseudomallei*. Ethical approval for this study was obtained from the Faculty of Tropical Medicine, Mahidol University, Thailand, and the Institutional Review Board Committee of the Angkor Hospital for Children.

Environmental sampling was performed during a 1-day period in January 2006. Four soil samples were collected from each of 10 different paddy fields around Siem Reap. Samples were collected on minor roads leading from route 6 (Thailand to Phnom Penh) up to 80 km southeast and 40 km northwest of Siem Reap. Sampling was performed and *B. pseudomallei* recovered as previously described (*8*). Susceptibility testing was performed by disk diffusion assay for ceftazidime, imipenem, and doxycycline, and by E test for trimethoprim-sulfamethoxazole. Interpretive standards were based on NCCLS guidelines (*9*).

Serum samples were obtained from 968 children, of whom 528 (54.5%) were male. The number of samples collected per year group is shown in the Figure. Fewer serum samples were collected from children 15 and 16 years of age because only a small number of samples were available from these age groups. A total of 159 children (16.4%) had a detectable IHA titer; values ranged from 10 to 10,240 (median 10, interquartile range [IQR] 20–640). Females were more likely to have a detectable IHA titer than males (86/440 females [19.5%] vs. 73/528 males [13.8%], p = 0.02), but the distribution of titer values in the population of positive female children was not significantly different from that in the population of positive male children. The proportion of children with any detectable IHA titer rose with age (Figure). An IHA titer >160 is used in some centers in Thailand to support a diagnosis of melioidosis in patients with clinical features consistent with this diagnosis. Sixty-three children (6.5%) had an IHA titer >160.

**Figure:**
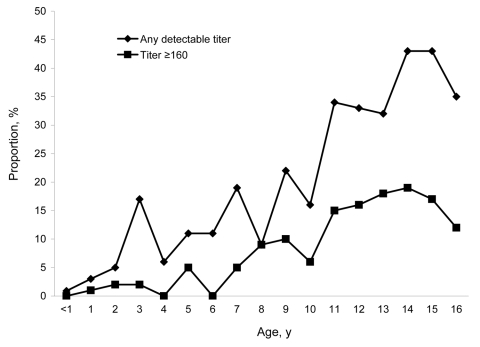
Indirect hemagglutination assay (IHA) titer for 968 children living in Siem Reap, Cambodia. The number of children tested in each year group is shown. The number of children by age group follows: <1 y, 106 children; 1 y, 98 children; 2 y, 93 children; 3 y, 54 children; 4 y, 50 children; 5 y, 62 children; 6 y, 55 children; 7 y, 57 children; 8 y, 44 children; 9 y, 49 children; 10 y, 50 children; 11 y, 41 children; 12 y, 49 children; 13 y, 66 children; 14 y, 54 children; 15 y, 23 children; 16 y, 17 children.

*B. pseudomallei* was isolated from 12 (30%) of 40 soil samples taken in 6 (60%) of 10 rice fields; 1 rice field was positive for all 4 samples, 3 fields were positive for 2 samples, and 2 fields were positive for 1 sample. CFU of *B. pseudomallei* per gram of soil ranged from 1 to 5,000 (median 90 CFU/g, IQR 20–250 CFU/g). *B. thailandensis,* a highly related but usually nonpathogenic organism, was isolated from 1 sample at a concentration of 5,000 CFU/g of soil. All 12 *B. pseudomallei* isolates were susceptible to ceftazidime, imipenem, amoxicillin-clavulanate, chloramphenicol, doxycycline, and co-trimoxazole.

## Conclusions

Detection of *B. pseudomallei* antibodies in 16% of Cambodian children is consistent with environmental exposure to this pathogen. The proportion of children with detectable antibodies is lower than that of children in adjacent northeast Thailand (*10*), although the overrepresentation of children in the first 3 years of life in this study could result in a lower comparative figure. The residents of both regions are predominantly agricultural workers and their families, and it seems unlikely that Cambodian children would have a lower level of environmental exposure. The median *B. pseudomallei* colony count of 90 CFU/g soil was lower compared with a reported figure of 230 CFU/g soil in northeast Thailand (*8*), which suggests that the bacterial inoculum present during a given exposure may be lower in Cambodia than Thailand. However, a direct link between environmental bacterial biomass and the rate of seropositivity in the healthy population remains unproven. Seropositivity using IHA is a crude surrogate for good blood culture–supported clinical epidemiology and case detection, although it provides a strong indirect indication of the presence of human melioidosis as a clinically important disease in Cambodia. It also underlines the need for building diagnostic microbiology capacity across rural Southeast Asia.
